# Vaccines to prevent bacterial sexually transmitted infections: Promise, progress, and public health potential

**DOI:** 10.1371/journal.pmed.1004849

**Published:** 2025-12-09

**Authors:** Sami L. Gottlieb, Helen Rees, Remco P. H. Peters

**Affiliations:** 1 World Health Organization, Geneva, Switzerland; 2 Wits RHI, Johannesburg, South Africa

## Abstract

Controlling transmission of the bacterial sexually transmitted infections (STIs) gonorrhoea, chlamydia and syphilis has proven difficult in recent years, and new solutions are needed. In this Perspective, Sami Gottlieb and colleagues discuss the promise and progress in the development of vaccines against bacterial STIs, highlighting the need for innovative research, expedited clinical development, and increased investment.

## Teaser

Asymptomatic transmission, inequitable access to diagnostics, and rising antimicrobial resistance are major barriers to controlling the bacterial sexually transmitted infections (STIs) gonorrhea, chlamydia, and syphilis. Developing vaccines against these infections has therefore become a key STI research priority, requiring innovative research, expedited clinical development, and increased investment.

The sexually transmitted infections (STIs) *Neisseria gonorrhoeae* (gonorrhea), *Chlamydia trachomatis* (chlamydia), and *Treponema pallidum* (syphilis) continue to pose an unacceptable global health burden. The World Health Organization (WHO) estimated 82 million new cases of gonorrhea and 129 million new cases of chlamydia worldwide among 15–49-year-olds in 2020, and 8 million new cases of syphilis in 2022 [1]. The resulting disease consequences disproportionately affect women and their newborns, particularly in low- and middle-income countries (LMICs) with higher prevalence and limited testing resources. Gonorrhea and chlamydia are important causes of female infertility, and untreated maternal syphilis has high fetal and neonatal fatality. Despite ongoing efforts, the global burden has changed little over the past decade, and cases are rising in some regions [1].

In the face of these trends, WHO has highlighted vaccine development for these pathogens as a key STI research priority [2,3]. Although gonorrhea, chlamydia, and syphilis are all curable infections, several biological, behavioral, and programmatic factors hinder control efforts. Major barriers include: high rates of asymptomatic infection; lack of feasible, affordable diagnostics for low-resource settings (gonorrhea, chlamydia); declining condom use; increasing antimicrobial resistance (AMR) (gonorrhea); and fragmented, underfunded programs [1,2,4]. Thus, vaccines may represent the only sustainable solution for addressing these STIs.

Gonococcal and chlamydial infections cause similar clinical syndromes and disease outcomes. The most serious reproductive consequences occur when cervical infection ascends to the upper genital tract to cause pelvic inflammatory disease (PID). Subsequent scarring can lead to chronic pelvic pain, ectopic pregnancy, and infertility. The precise burden of infertility from these infections is unknown, but comprehensive estimates from the UK suggest that five cases of infertility result from every 1,000 chlamydial infections [5]. Rates are likely similar for gonorrhea and potentially higher in LMICs with more limited diagnostics and treatment. With ~100 million estimated gonococcal and chlamydial infections among women each year [1], the number of resulting infertility cases could be substantial.

The most imminent threat to controlling gonococcal infection is AMR. Resistance of *N. gonorrhoeae* to third-generation cephalosporins—the last effective first-line drugs—is increasing [1]. As *N. gonorrhoeae* has rapidly developed resistance to every antimicrobial class used to date, new antibiotic options may offer only short-term benefits. The growing risk of potentially untreatable gonorrhea underscores the urgent need for gonococcal vaccines.

*N. gonorrhoeae* exhibits frequent antigenic variability and does not induce natural immunity. As a result, vaccine development received limited attention until an intriguing epidemiologic observation. In a few countries fighting meningitis due to *Neisseria meningitidis* serogroup B (MenB, a bacterium closely related to *N. gonorrhoeae*), mass vaccination campaigns were associated with declines in reported gonorrhea cases [6]. Studies evaluating this potential cross-protection suggested that MenB vaccines containing outer membrane vesicles (OMVs) could moderately reduce acquisition of gonococcal infection. Meta-analyses of eight observational studies and one small randomized controlled trial (RCT) estimated pooled vaccine effectiveness at 30%–35% [6,7]. In 2025, evaluation of an adolescent MenB OMV vaccination programme in South Australia explored duration of protection against gonorrhea, estimating 42% protection within 5 years but no significant effect thereafter [8].

Multiple RCTs are currently underway to evaluate the efficacy of the commercially available OMV-based vaccine 4CMenB (Bexsero, GSK) in preventing gonorrhea (Table 1). Confirmed efficacy, even if moderate, could provide a much-needed prevention option. Modeling suggests that even vaccines with ~30% efficacy could have substantial benefits in reducing gonorrhea population prevalence and potentially AMR, especially with high uptake among at-risk populations [9].

**Table 1:** Randomized controlled trials of bacterial STI vaccine candidates in the clinical pipeline*.

Trial number	Phase/type of study	Study name	Location, number of participants	Population	Status
*Neisseria meningitidis* serogroup B vaccines for preventing gonococcal infection					
ACTRN12619001478101	Phase 3 randomized controlled trial (RCT) efficacy, immunogenicity	MenGO: Does the licensed meningococcal vaccine Bexsero provide cross-protection against gonorrhea?	Australia 130	Men who have sex with men (MSM)	Results expected late 2025
NCT04415424	Phase 3 RCT efficacy, immunogenicity	GoGoVax: Efficacy study of 4CMenB (Bexsero) to prevent gonorrhea infection in gay and bisexual men	Australia 730	MSM	Results expected late 2025
NCT05766904	RCT efficacy	Efficacy trial on meningococcal B vaccine for preventing gonorrhea infections	Hong Kong 150	MSM	Results expected late 2025
NCT04350138	Phase 2 RCT efficacy	Safety and efficacy study of meningococcal group B vaccine rMenB+OMV NZ (Bexsero) to prevent gonococcal infection	USA, Thailand, Malawi 2,200	Adults	Results expected mid-2026
NCT06446752	Phase 3 RCT efficacy	BIYELA: Efficacy of Bexsero in preventing gonococcal infection among South African *cis-*gender women	South Africa 1,100	Cis-gender women	Results expected late 2026
NCT05294588	RCT efficacy in controlled human infection challenge model	Efficacy of immunization with 4CMenB in preventing experimental urethral infection with *Neisseria gonorrhoeae*	USA 140	Men	Results expected 2028
*N. gonorrhoeae*-specific vaccines					
NCT05630859	Phase 1/2 RCT safety, efficacy, immunogenicity	Safety and efficacy of GSK *N. gonorrhoeae* GMMA (NgG) investigational vaccine when administered to healthy adults 18–50 years of age	Multi-country 1,004	Adults	Data collection complete in May 2025. Results not yet reported, but developer reports product will be removed from pipeline (https://www.gsk.com/media/lkklkfgi/q3-2024-pipeline-assets-and-clinical-trials-report.pdf)
*Chlamydia trachomatis* vaccines					
NCT06891417	Phase 1/2 RCT safety, efficacy, immunogenicity	Phase 1/2 study of *C. trachomatis* mRNA vaccine in adults aged 18–29 years	Australia 1,560	Adults	Results expected 2028

*as of 18th November 2025.

Based on existing observational evidence, some jurisdictions have already begun implementing 4CMenB immunization for gonorrhea prevention. In August 2025, the UK began offering 4CMenB through sexual health clinics to primarily gay, bisexual, and other men who have sex with men with high infection risk [10]. The UK National Immunization Programme already includes 4CMenB for meningitis, and UK-specific modeling predicts this approach could reduce population-level gonorrhea incidence and be cost-effective, given the country’s concentrated epidemic [9]. Real-world follow-up will be essential to understand vaccine uptake and impact [10].

Most LMICs with the highest gonorrhea burden do not use MenB vaccines—either due to low incidence of MenB disease or because the vaccine is too expensive [11]. Proven efficacy against gonococcal infection, including among women in Southern Africa with the greatest risk of disease sequelae (Table 1), could tip the balance with respect to cost-effectiveness to justify broader use of MenB vaccines, whether for gonorrhea alone, or for both conditions.

Importantly, immunological data from these trials will be valuable for developing a more efficacious gonococcal-specific vaccine. Although several candidates are in preclinical stages, only one gonococcal-specific vaccine (NgG, GSK) has recently been in clinical development, but it is reportedly not being taken forward (Table 1). Insights from the 4CMenB RCTs, including mechanisms and duration of partial protection, can guide purposeful design of gonococcal-specific vaccines.

*C. trachomatis* vaccines have long been sought, as genital chlamydia is the most prevalent bacterial STI worldwide and a key preventable cause of female infertility. Vaccines have also been pursued for trachoma, an ocular infection caused by different *C. trachomatis* serovars and a major infectious cause of blindness. The natural history of genital *C. trachomatis* infection contributes to population-wide transmission, as asymptomatic infection can persist for months in women, and repeat infections are common. Despite longstanding screening and treatment programs in several high-income countries, reducing population-level chlamydia prevalence has proven difficult. Thus, developing chlamydial vaccines remains an important goal.

Even with evidence of some naturally occurring immunity and years of research into chlamydial immunobiology, by early 2025, only one candidate had been in clinical development in the past decade. The recombinant chlamydial major outer membrane protein vaccine CTH522 (Statens Serum Institute), designed for both genital and ocular chlamydia, first completed Phase 1 trials in 2017 and demonstrated safety and strong humoral and cellular immune responses [12]. However, this candidate has not yet entered phase 2 trials. Recent innovations in mRNA platform technology have led to the first real advance in years. A new chlamydial vaccine candidate based on the mRNA platform (Chlamydia mRNA, Sanofi) received FDA fast-track designation and began Phase 1/2 clinical evaluation in March 2025 (Table 1).

Such advances provide hope for a long-term goal: A combined gonococcal-chlamydial vaccine. Co-infection rates are high, and the greatest potential benefit of vaccines for both pathogens lies in preventing upper genital tract complications in women. Better diagnostic tools for PID and related sequelae are needed to better quantify the disease burden these vaccines could avert and inform trial endpoints [2].

Syphilis also warrants consideration for vaccine development, despite lower incidence than the other bacterial STIs, due to its continuing large disease burden [1]. Globally, syphilis causes an estimated 390,000 adverse birth outcomes each year, including 220,000 stillbirths and neonatal deaths, and congenital syphilis is resurging in some settings [1]. These outcomes are largely preventable through timely antenatal screening and treatment, yet syphilis control has faced inadequate program implementation and recurring benzathine penicillin treatment shortages [2].

*T. pallidum*, the syphilis bacterium, has few surface-exposed proteins, making it hard for the immune system and vaccines to target [13]. Experimental vaccines targeting outer membrane proteins have demonstrated partial protection in rabbits, but none have reached human studies. Current research focuses on identifying vaccine targets and finding suitable adjuvants and delivery platforms. While efforts are redoubled to strengthen existing interventions and find new treatment options [1,2], work should continue in parallel on syphilis vaccines.

Vaccines against gonorrhea, chlamydia, and syphilis could provide lasting solutions for these often-neglected pathogens. The global need remains high, as epidemiological data indicate limited progress—and, in some regions, worsening trends—in bacterial STI control. A decade ago, WHO, the US National Institutes of Health, and partners outlined a global STI vaccine roadmap [3]. Since then, progress in studying MenB vaccine cross-protection against gonorrhea has led to multiple RCTs, which will not only clarify the prevention potential of existing MenB vaccines, but will also guide the design of more efficacious gonococcal-specific vaccines. The first new chlamydial vaccine candidate entering clinical trials in years has highlighted how new technologies—in this case, mRNA platforms—can be harnessed to revitalize STI vaccine development. To build on this momentum, we need stronger data on disease burden and cost-effectiveness, expedited discovery and clinical development using tools like artificial intelligence [14], and early planning for vaccine introduction [3]. Sustained focus and increased investment are crucial for advancing vaccine development for gonorrhea, chlamydia, and syphilis – priority interventions for global sexual and reproductive health.

## Abbreviations

AMR: antimicrobial resistance
LMIC: low- and middle-income countries
OMV: outer membrane vesicle
PID: pelvic inflammatory disease
RCT: randomized controlled trial
STI: sexually transmitted infection
WHO: World Health Organization
